# Efficient “Communication through Coherence” Requires Oscillations Structured to Minimize Interference between Signals

**DOI:** 10.1371/journal.pcbi.1002760

**Published:** 2012-11-08

**Authors:** Thomas E. Akam, Dimitri M. Kullmann

**Affiliations:** University of College London Institute of Neurology, London, United Kingdom; Université Paris Descartes, Centre National de la Recherche Scientifique, France

## Abstract

The ‘communication through coherence’ (CTC) hypothesis proposes that selective communication among neural networks is achieved by coherence between firing rate oscillation in a sending region and gain modulation in a receiving region. Although this hypothesis has stimulated extensive work, it remains unclear whether the mechanism can in principle allow reliable and selective information transfer. Here we use a simple mathematical model to investigate how accurately coherent gain modulation can filter a population-coded target signal from task-irrelevant distracting inputs. We show that selective communication can indeed be achieved, although the structure of oscillatory activity in the target and distracting networks must satisfy certain previously unrecognized constraints. Firstly, the target input must be differentiated from distractors by the amplitude, phase or frequency of its oscillatory modulation. When distracting inputs oscillate incoherently in the same frequency band as the target, communication accuracy is severely degraded because of varying overlap between the firing rate oscillations of distracting inputs and the gain modulation in the receiving region. Secondly, the oscillatory modulation of the target input must be strong in order to achieve a high signal-to-noise ratio relative to stochastic spiking of individual neurons. Thus, whilst providing a quantitative demonstration of the power of coherent oscillatory gain modulation to flexibly control information flow, our results identify constraints imposed by the need to avoid interference between signals, and reveal a likely organizing principle for the structure of neural oscillations in the brain.

## Author Summary

Distributed regions of mammalian brains transiently engage in coherent oscillations, often at specific stages of behavioral or cognitive tasks. This activity may play a role in controlling information flow among connected regions, allowing the brain's connectivity structure to be flexibly reconfigured in response to changing task demands. We have used a computational model to investigate the conditions under which oscillations can generate selective communication through a mechanism in which the excitability of neurons in one region is modulated coherently with a firing rate oscillation in another region. Our results demonstrate that this mechanism is able to accurately and selectively control the flow of signals encoded as spatial patterns of firing rate. However, we found that the requirement to avoid interference between different signals imposes previously unrecognised constraints on the structures of oscillatory activity that can efficiently support this mechanism. These constraints may be an organizing principle for the structured oscillatory activity observed *in vivo*.

## Introduction

Task-dependent changes in the power and inter-region coherence of oscillatory network activity are observed in many brain regions and behavioral tasks [1]–[3]. A possible function of such activity is to modulate functional connectivity among anatomically connected regions [4]–[6]. This may play an important role in cognition by allowing the structure, and hence function, of brain networks to be dynamically reconfigured in response to different task demands.

The ‘communication through coherence’ (CTC) hypothesis [5], [7] proposes that selective communication is achieved through coherence between firing rate oscillation in the sending region and oscillatory gain modulation in the receiving region. This could, theoretically, allow a network to respond selectively to a task-relevant ‘target’ signal while ignoring other distracting inputs. However, the conditions under which accurate selective communication can be achieved by this mechanism remain unclear. Intuitively, it appears likely that the accuracy with which a target signal can be filtered from distractors will depend on how they differ with respect to the oscillatory modulations of their firing rates. Clearly, if target and distracting inputs have the same modulation, coherent gain modulation cannot separate them; but in what way and to what extent must their modulations differ in order for the target signal to be accurately recovered? Understanding which structures of oscillatory activity can support accurate selective signal transmission is an important step in evaluating whether activity patterns observed in vivo are consistent with their proposed functional role in routing information flow. Despite extensive experimental [8]–[10] and computational work [11]–[14], it remains unclear under what conditions the CTC mechanism could allow a network to distinguish among converging population-coded signals, and how its performance depends on the structure of their oscillatory modulations.

We recently developed a convergent pathway model to investigate oscillatory routing of information flow [15]. In the present study we have used a similar paradigm to address these questions. Our results indicate that where inputs are distinguished by the frequency, phase or amplitude of their modulations they can be readily separated by coherent gain modulation, but that attempting to separate inputs that oscillate incoherently in the same frequency band results in greatly increased noise and reduced communication accuracy. Additionally, the oscillatory modulation of the target input must be strong to ensure a high signal to noise ratio relative to stochastic spiking of individual neurons. These constraints on patterns of activity that efficiently support flexible routing of information may be an organizing principle for the rich structures of neural oscillations observed *in vivo*.

## Materials and Methods

For clarity we first describe the model with minimal use of equations and explain the rationale behind the choices made in its design. We then detail the equations and mathematical methods used to generate the results.

### Model overview

We modeled a convergent pathway in which multiple input networks converged to a single receiving network. The task required of the model was to selectively route a behaviorally relevant signal encoded in an input network (the ‘target’ input) to the receiving network, while ignoring simultaneously active distracting inputs in other converging networks. While not specifically a model of any particular region, this network design minimally recapitulates many converging cortical and sub-cortical pathways where selective information flow may be required. Input selectivity was achieved by oscillatory gain modulation in the receiving network, coherent with oscillatory modulation of the firing rate of the target input network. To evaluate how accurately this gain modulation could filter the target input from distractors, the output of the receiving network was integrated over time and decoded to produce an estimate of the stimulus encoded by the target input.

### Input networks

Each input network modeled a local population of neurons representing a separate one-dimensional circular variable using a firing rate population code (e.g. a cortical hypercolumn). The average firing rates of individual neurons were given by bell-shaped tuning curves with respect to the stimulus orientation. Spike times in each neuron were determined by a Poisson process whose instantaneous rate could be simultaneously modulated for all neurons within a given network to simulate a population oscillation. This modulation was modeled as a Von Mises function of the phase of the oscillation, characterized by a modulation strength and frequency. As network oscillations in vivo are irregular in frequency and amplitude, we allowed the instantaneous strength and frequency to fluctuate around their mean values. These fluctuations were modeled as low-pass filtered Gaussian white noise. The resulting activity was consistent with in vivo data showing irregular spiking of single units [16]–[18] during sparsely synchronized oscillatory activity [19].

### Receiving network

We initially considered a situation in which an external control input synchronized activity in the target and receiving networks, such that the oscillatory modulation 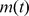 of the target input firing rate was known by the receiving network. The receiving network must exploit this known temporal structure to generate a pattern of gain modulation that separates target from distracting inputs, and hence recover the spatial population code representing the target stimulus. We later compare the performance of models with and without such an external synchronizing input.

Physiologically, gain modulation could be achieved by local interneuron circuitry modulating the distribution of membrane potentials, degree of shunting inhibition [20], or synaptic noise [21] experienced by the principal neurons of the receiving network. Oscillatory gain modulation coherent with the target input could then be generated by driving such interneuron circuitry with the oscillating external control signal. Although this arrangement could, in principle, be implemented in a biophysical model, we required a model of the receiving network that could be optimized to generate the temporal pattern of gain modulation that *best* separated the target from distracting inputs. If the receiving network was not optimized, the results would be uninformative about the performance of the mechanism in general and would only shed light on the specific implementation.

Two obstacles made optimizing a biophysical model intractable. Firstly, we do not know *a priori* what waveform the optimal gain modulation should take for a given temporal pattern of input activity. Secondly, it remains incompletely understood how neuronal and network parameters determine the response of networks to temporally structured inputs. Therefore, even if we knew what temporal pattern of activity the interneurons in the receiving network must generate in response to a given control signal, it would not be straightforward to design such a network with the appropriate dynamics. Given these difficulties with optimizing a biophysical model we instead developed an algorithmic description of the receiving network's operation that could be optimized with respect to the mean squared error of the target stimulus estimates decoded from its output.

The receiving network model consisted of two components. The first component was a layer of projection units, which received convergent population-coded signals from the input networks and formed the output of the receiving network. Each unit in the projection layer represented a population of cells innervated by neurons with similar orientation preferences in each input network and whose output was an analog firing rate signal. The output 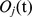 of unit 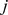 was given by:
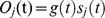
where 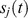 was the spike input received by the unit and 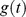 was a temporal pattern of gain modulation (see below). We allowed the gain to take both positive and negative values (corresponding to net excitatory and inhibitory output respectively) such that spikes arriving during periods of negative gain contributed negatively to the integrated output. Though we do not explicitly model the circuitry, a simple micro-circuit supporting positive and negative net gain would be a pathway where excitation is balanced by feed forward inhibition, with gain modulation acting on the inhibitory neurons.

The second component of the receiving network represented the local interneuronal circuitry, which received the oscillating top-down control signal 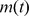 and converted this into a temporal pattern of gain modulation 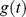, which was applied uniformly to all projection units. In an optimized filtering network the dynamics of this circuitry must be such that the pattern of gain modulation generated in response to a given control input is that which best filters the target from distracting inputs. Rather than model these dynamics directly we instead represented them as a filtering process. Optimizing the receiving network then became a problem of finding the filter that transformed the firing rate modulation of the target into the gain modulation that best separated the target from distracting inputs. This problem is closely analogous to that of matched filtering in the engineering literature in which a target signal of known waveform must be detected against a background of noise.

We initially considered gain modulations that were linearly filtered versions of the firing rate modulation of the target input. For this model, we could rapidly optimize the frequency response of the filter using gradient descent on training data (see Materials and Methods), allowing us to explore the parameter space of input activity patterns. We then verified that our key results obtained for this linear model held when we allowed the gain modulation to be an arbitrary function of the firing rate modulation of the target input.

### Decoding

The output of the receiving network was integrated over 100 ms to give a spatial pattern of activity. This was then decoded to produce an estimate of the target stimulus. We therefore only considered the information contained in the average firing rates of the receiving network output units over the integration window. We report the lower bound on the Fisher information given by the reciprocal of the mean squared error of the stimulus estimates. Locally optimal linear estimators (LOLEs) were used for decoding. These decoders were sufficiently simple to permit optimization of the temporal filtering with respect to the root mean squared error of decoded stimulus estimates using gradient descent (see Experimental Procedures). Under many noise distributions these decoders perform close to optimally, as indicated by the minimal difference in performance when compared with more sophisticated non-linear methods [22], [23]. These decoders are, moreover, biologically plausible as their performance corresponds to that of de-noising by networks implementing line attractor dynamics [24], [25].

### Model equations


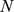
 input networks, each consisting of 10,000 Poisson neurons, represented independent orientation variables 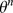. The firing rate of the 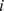
th neuron in input network 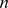 was given by:
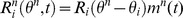
Where 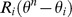 is a firing rate tuning curve with respect to stimulus orientation (range 0–180°) and 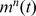 is an oscillatory firing rate modulation:
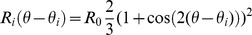
Where 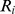 is the firing rate of the 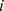
th neuron, 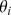 is the neurons preferred orientation and 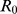 is the average firing rate across the population.

The oscillatory modulation was a Von Mises function of the oscillation phase 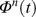:
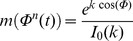
Where 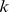 is a concentration parameter that determines how tightly synchronized the activity is, and 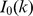 is the modified Bessel function of order 0 which normalizes the modulation such that its average value over time is 1. A sinusoidal modulation was also used where indicated, given by the equation:
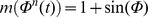
To model the irregularity of network oscillations we allowed the oscillation angular frequency 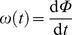 and concentration parameter 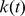 to fluctuate around their mean values 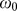 and 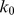. These fluctuations were modeled as:
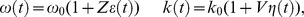
where 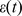, 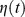 were low-pass filtered Gaussian white noise with amplitude 1 and a cut-off frequency of 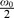. The variability of the frequency and amplitude respectively were therefore determined by 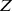 and 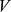, which were the standard deviation of fluctuations divided by the mean value.

Where we report synchronization strength, we use the following measure:
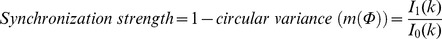
Where 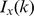 is the modified Bessel function of order 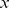. This measure is 1 if all spikes occur at the same phase and 0 if the firing rate is equal at all phases.

The combined input spike rate 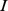 impinging on the receiving network was the sum of activity in all input networks:
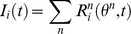
The combined spike input 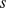 was:
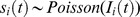
The receiving network consisted of a layer of 8 units, each of which received spike input from neurons with similar orientation preference in each input network. The range of orientation preference from 0° to 180° was divided into 8 equal width bands and neurons in each input network with orientation preference in a given band projected to the same unit in the receiving network. The combined input 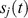 to unit 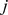 in the receiving network was:
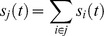
Where the sum was over those units 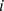 in the input networks that projected to unit 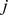 in the receiving network.

The output 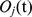 of unit 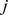 in the receiving network was:
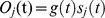


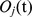
 was integrated over time 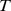 to produce a spatial pattern of activity 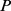:
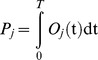
An estimate 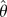 of the stimulus encoded in the target input network was decoded from the integrated activity using a LOLE.
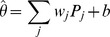
where 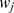 are the weights for each unit of the receiving network and 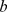 is a constant.
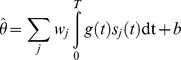
The simulations were performed in discrete time with a resolution of 1 ms, such that the integral over time was computed as a sum over time bins:
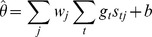
where 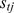 is the spike count received by unit 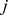 in time bin 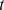.

### Optimizing the receiving network

Except where specified otherwise, the gain modulation 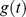 was a linearly filtered version of the modulation of the target input 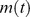. For this model we optimized the receiving network by using gradient descent to find the frequency response of the filter (the gain and phase shift as a function of frequency) which optimized decoding accuracy with respect to the variance of the stimulus estimates.

We used Plancherel's theorem to express 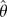 in terms of the discrete Fourier transforms (DFTs) 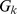 and 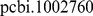 of the gain modulation 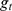 and spike input 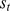. (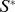 indicates complex conjugate, 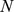 is the number of components in the Fourier transforms).
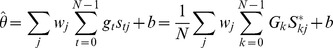
As the gain modulation and spike input are both real valued, the imaginary parts of 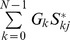 at positive and negative frequencies cancel and only the real parts contribute to the sum.
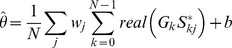
Also, 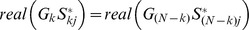 so the second half of the sum is redundant:
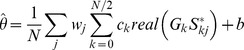
where 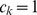 for 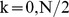, 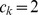 for 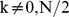.

We expressed the Fourier transform of the gain modulation 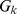 as the product of the frequency response of the filter 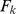 and the Fourier transform 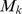 of the target inputs firing rate modulation. The frequency response is a complex valued function of frequency where 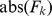 is the gain of the filter at frequency k and 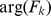 is the phase shift.
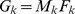
We can then express the stimulus estimate as:
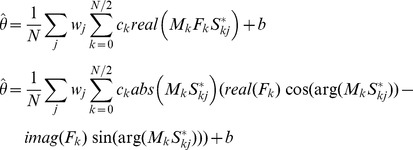
We define 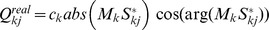 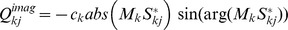


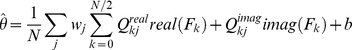
This can be written in vector notation as:
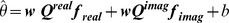
Where 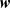 is a row vector whose components are the weights of the LOLE, 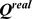 and 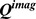 are matrices whose components are 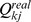 and 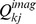, and 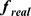 and 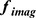 are column vectors whose components are respectively the real and imaginary parts of the filter frequency response 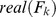 and 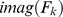. To further simplify the expression we concatenate the vectors 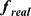 and 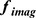 to make a single vector 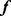 containing both the real and imaginary parts of the filter frequency response and concatenate the matrices 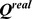 and 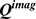 to make a single matrix 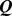. We can now express the decoded stimulus estimate as:
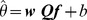
To use gradient descent to find the optimal LOLE weights and filter frequency response we define a cost function and calculate the gradient with respect to it. We define the cost function as the squared error between the true stimulus value and the decoded estimate, averaged over a training set of data:
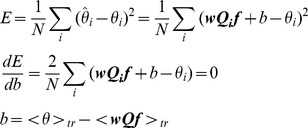
Where 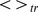 indicates the average over the training set.
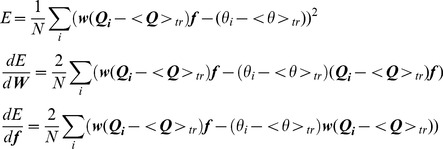



### Model parameters

The following default parameter values were used except where stated otherwise:

Average neuronal firing rate 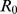 = 5 Hz, Synchronization strength = 0.5, Modulation frequency = 50 Hz, Amplitude variability parameter 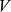 = 0.1.

The frequency variability parameter 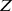 was set to 0.1 for narrowband oscillations and 0.3 for broadband oscillations. Narrowband oscillations were used except those where distractors oscillated incoherently in the same frequency band as the target.

### Simulations and analysis

The following procedure was performed to establish decoding accuracy for each set of input parameters. All simulations were performed in MATLAB. A training set and a test set of input activity were generated, each consisting of 5000 samples of 100 ms each. In each set, half of the samples had target stimulus orientation 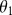 and the other half 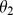. The separation 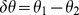 was chosen iteratively such that 75–80% of samples were correctly classified from the decoded stimulus estimates. The orientation of stimuli encoded in the distracting input networks were uniformly randomly distributed in all samples. To reduce spectral leakage due to finite integration times, we applied a Hann window to the spike activity in each sample. The weight vectors for the LOLE and the frequency response of the filter were optimized using a two-stage gradient descent procedure. Firstly we used gradient descent to find the filter frequency response 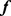 that minimized the mean squared error between the output of the units comprising the receiving network and the firing rate each unit received from neurons in the target input network. This gradient descent stage was initialized with all components of the filter frequency response set to zero. We then performed gradient descent simultaneously on the LOLE weights 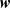 and the filter frequency response 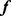 to minimize the mean squared error of target stimulus estimates, using the gradients calculated above. This second stage of the gradient descent was initialized with the filter frequency response found in the first gradient descent and with the weights of the LOLE set to zero. We used this two stage procedure because it converged much more rapidly than initializing the simultaneous gradient descent for 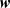 & 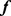 with small random weights. To prevent over-fitting we evaluated the mean squared error for the test set and halted gradient descent when this started to rise. We evaluated the mean and variance of the stimulus estimates for both orientations on the test set:
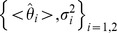
The lower bound on the Fisher information was given by:
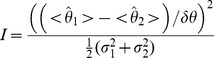
When examining decoding accuracy for integration times up to 1,000 ms, the gradient descent took a very long time because of the larger number of weights to be fitted. We had observed in other simulations that the amplitude of the filter frequency response 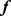 was consistently zero at high frequencies where there was minimal power in the target modulation. For all simulations in this figure we therefore set the filter frequency response to zero for frequencies above 3 times the target modulation frequency, reducing the number of weights that had to be fitted. We verified for a subset of simulations that this minimally affected decoding accuracy.

We also allowed the gain modulation to be an arbitrary function of the modulation of the target input. To do this we generated training and test sets as described above, but instead of generating a different modulation of the target input network for each sample, we used the same modulation of the target input network while generating different modulations for the distractors. Instead of optimizing the filter parameters 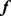 that transformed the modulation of the target input into the gain modulation, we directly fitted the gain modulation 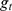 using gradient descent. To do the gradient descent we rewrite the equation for the stimulus estimate in vector form:
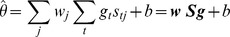
Where 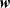 is a row vector with components 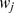, 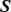 is a matrix with components 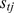 and 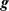 is a column vector with components 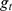. As this has identical form as 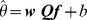, we can use the gradient descent procedure described above to find the gain modulation 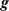 that minimizes the mean squared error of target stimulus estimates. Decoding accuracy varies somewhat depending on the precise waveform of the modulation of the target, so we repeated the procedure 100 times using different instances of the gain modulations of the target input, and report the mean and standard deviation of the decoding accuracy over these different modulations.

## Results

### Selective communication by coherent gain modulation

**Figure 1:**
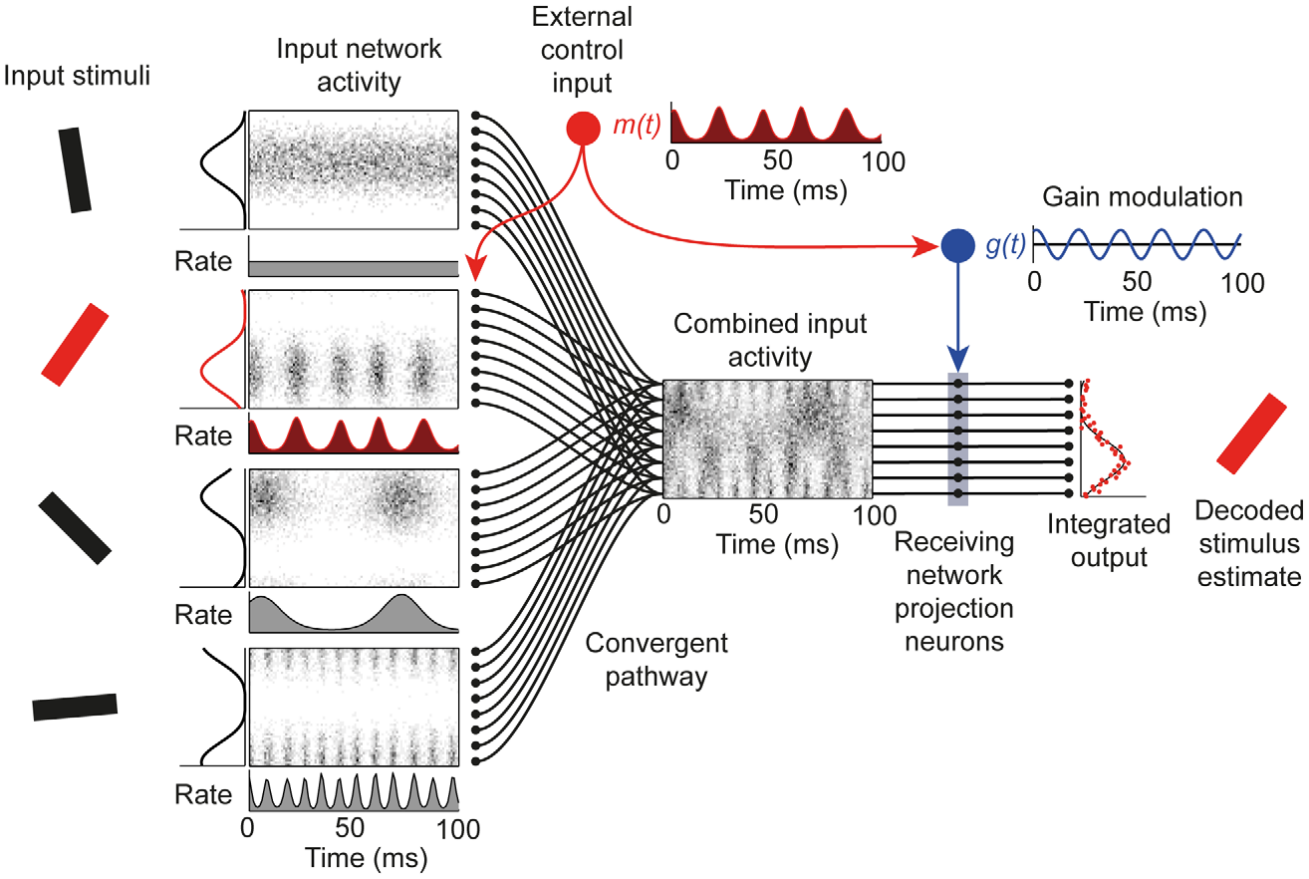
Selective communication by coherent gain modulation. Independent orientation stimuli are represented in separate input networks as population codes with bell-shaped firing rate tuning curves. These input networks converge to provide a combined input to the receiving network. To selectively route the information encoded in one input network (the ‘target’ input) to the output of the receiving network, a top-down control signal imposes an oscillatory modulation on the target network firing rate and a coherent oscillatory gain modulation in the receiving network. The output of the receiving network is integrated over time to produce a spatial pattern of activity, which is decoded to produce an estimate of the target stimulus.

Figure 1 illustrates the power of coherent gain modulation to filter an oscillating population-coded target signal from distractors, and hence achieve selective communication. Though four different inputs of equal average firing rate converge on the receiving network, the integrated output reflects only the spatial pattern of activity in the target input, and the stimulus encoded by this input can be accurately decoded from the output.

How does this selection occur? The effective gain for each converging pathway is determined by the overlap between the input's firing rate modulation and the gain modulation in the receiving region, averaged over the integration window. In this case the gain modulation in the receiving region is approximately sinusoidal and in phase with the target input (2^nd^ input network from the top in Figure 1). The target input contributes strongly to the integrated output because periods of high firing rate occur concurrently with large positive gain. Because the average gain is zero, the distractor whose units fire asynchronously, without any population firing rate modulation (first input network), contributes minimally to the integrated output. Likewise, for distractors oscillating at frequencies well separated from the target (3^rd^ and 4^th^ networks) the average overlap between firing rate modulation and the gain modulation is very close to zero, and hence they also contribute minimally to the integrated output.

Mathematically, the signal from distracting inputs is rejected because their firing rate modulations are either zero or orthogonal to the gain modulation in the receiving region. For accurate selective communication to be achieved by this mechanism, there must exist a pattern of gain modulation that is strongly driven by the modulation of the target input but close to orthogonal to the modulations of the distracting inputs. As we will see in the next section, this imposes constraints on the structure of oscillatory activity in the converging inputs.

### Oscillation structure determines communication accuracy

**Figure 2:**
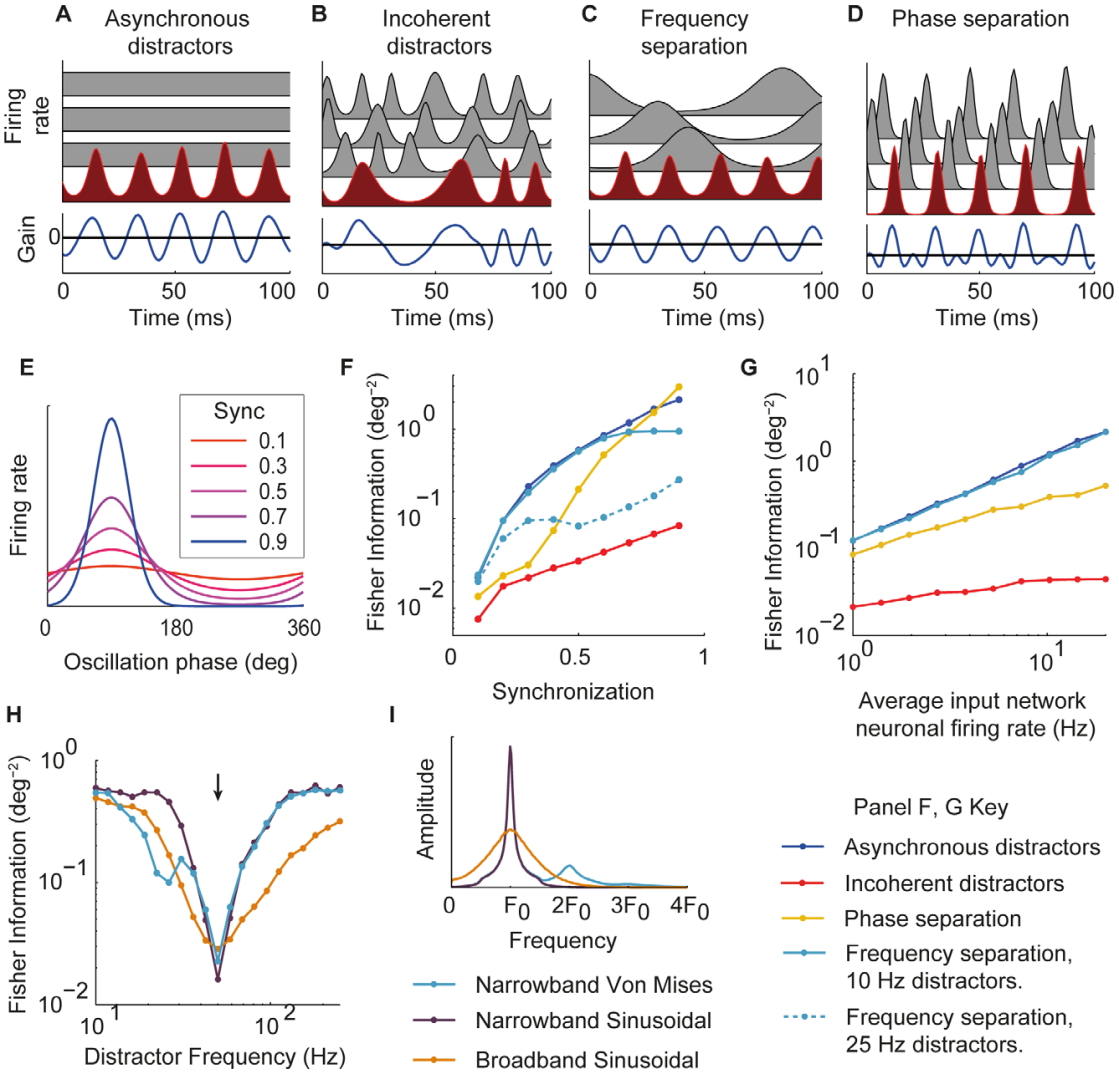
Oscillation structure determines communication accuracy. (*A*–*D*) Example firing rate modulation of the target (red) and distracting inputs (gray) over the 100 ms integration time. Gain modulation (blue) produced by the optimized receiving network. (*E*) Firing rate as a function of oscillation phase for synchronization strengths from 0.1–0.9. (*F*) Fisher information as a function of the synchronization strength of the target input for stimulus estimates decoded from receiving network output integrated over 100 ms. Distractor condition indicated by color as shown in key. (*G*) Comparison of Fisher information for asynchronous and incoherently oscillating distracting inputs as functions of firing rate of input networks. (*H*) Separation of target and distractors in frequency. Fisher information as function of oscillation frequency of distractor networks for narrowband (purple) and broadband (orange) sinusoidal oscillations and narrowband Von Mises oscillations (blue). Frequency of target input modulation (50 Hz) is indicated by black arrow. (*I*) Amplitude spectrum of oscillatory modulations for narrowband Von Mises modulation and narrow and broadband sinusoidal oscillations (F_0_ is oscillation center frequency).

We evaluated the accuracy of selective communication for four different structures of oscillatory activity in the input pathways (Figure 2A–D). We first considered a condition in which only the target input was oscillating while the units in the distractor networks fired asynchronously. The gain modulation produced by the optimized filtering network was near-sinusoidal, in phase with the oscillation in the target input (Figure 2A). Decoding accuracy depended strongly on the strength of oscillatory modulation of the target input (Figure 2F). Accuracy was high for strongly modulated input, dropping steeply as the oscillation strength decreased. We quantified the depth of modulation of the target input firing rate (‘synchronization’) using a metric that ranged from 0 for fully asynchronous activity to 1 if all spikes occur at the same phase of the modulation (see Materials and Methods, Figure 2E). Over the range of synchronization from 0.1 to 0.9, Fisher information increased by a factor of 95.7, with the majority of this increase occurring in the range from weak to moderate synchronization (Fisher information increased 26-fold when synchronization strength increased from 0.1 to 0.5, and 3.65-fold as it increased from 0.5 to 0.9). Weak target input modulation resulted in poor decoding accuracy because the signal read out by the receiving network was small relative to noise from stochastic spiking of distracting inputs. Across a wide range Fisher information increased with the average firing rate in the target and distractors (Figure 2G).

We next evaluated the proposal that changes in the inter-region coherence of oscillatory activity [5], [8], [10] (as distinct from changes in frequency or amplitude, or changes in a consistent phase relationship) could be used to switch on or off information propagation through a convergent pathway. In our model, this corresponds to distracting inputs oscillating irregularly in the same frequency band as the target (Figure 2B). A corollary of this CTC scheme is ‘non-communication through non-coherence’ [5] whereby absence of a reliable phase relationship between firing rate modulation in the sending target network and gain modulation in the receiving region prevents information transmission.

Fisher information was greatly reduced for pathways in which distracting inputs oscillated incoherently compared with pathways in which distracting inputs were asynchronous (Figure 2F,G). The relative performance in the two conditions depended on the firing rate of the input networks. For pathways with asynchronous distractors, information increased linearly, but with incoherently oscillating distractors, information increased sublinearly with the firing rate of the input networks (Figure 2G). For average firing rates of 1 Hz per neuron, the Fisher information was 5.7 times higher when distractors fired asynchronously than when they oscillated incoherently, and this ratio increased to 27.8 when the average firing rate was 10 Hz. Increasing the synchronization of oscillations in the input networks improved decoding accuracy (Figure 2F), but across all oscillation strengths accuracy was much higher for pathways with asynchronous distractors.

Two aspects of the input network activity were changed between the asynchronous and incoherent distractors conditions; the modulation of the distracting inputs but also the variability of the frequency of the target input which was narrow in the asynchronous distractors case but broad in the incoherent distractors case. To determine which of these changes degraded communication accuracy we evaluated a condition with broadband incoherent distracting inputs but narrowband modulation of the target input (Figure S1A), and a condition with asynchronous distractors but broadband modulation of the target input (Figure S1B). Changing from a narrowband to a broadband modulation of the target signal did not affect communication accuracy with either asynchronous or incoherent distractors (Figure S1C). Changing from asynchronous to incoherent distractors dramatically reduced communication accuracy for both narrowband and broadband modulation of the target input (Figure S1C). These results indicate that it is the incoherently oscillating distracting inputs that degrade communication accuracy.

The poor communication accuracy in the incoherent distractors condition can be understood by considering the overlap between the gain modulation in the receiving network and the firing rate modulation of the distractors. From cycle to cycle the distracting inputs will drift in and out of phase with the target input, and hence with the gain modulation in the receiving region. This causes large fluctuations in the effective gain for distracting inputs, a source of ‘overlap’ noise quite distinct from that due to stochastic spiking of individual neurons. Although we have measured the accuracy of signal estimation for a given integration time, an alternative effect of this additional source of noise is an increase in the integration time required to reach a given decoding accuracy when compared with asynchronous distracting inputs, i.e. a decrease in the rate of information transmission through the pathway.

The differential dependence of communication accuracy on firing rate in the asynchronous and incoherent distractors conditions can be understood by considering more closely the two sources of noise that degrade the stimulus estimate. Noise due to stochastic spiking of individual neurons occurs for both asynchronous and oscillating distractors, and becomes smaller relative to the signal as the firing rates of the input networks increase. Overlap noise, in contrast, occurs only for oscillating distractors and increases in proportion to the signal size with increasing input firing rates. With incoherently oscillating distractors, this second source of noise becomes dominant as the mean firing rate increases, and prevents a further increase in signal-to-noise ratio.

At very low firing rates, noise in the output of the receiving network is dominated by stochastic spiking of individual neurons. In this regime we found decoding accuracy to be comparable for asynchronous and incoherently oscillating distractors. In supporting information we evaluate the firing rate threshold above which overlap noise dominates (Text S1 and Figure S2). Above this threshold ‘non-communication through non-coherence’ results in severe signal degradation compared with schemes in which distracting inputs are asynchronous or separated from the target in frequency or phase (see below). This threshold is proportional to the oscillation frequency, but for physiological frequencies it is low relative to firing rates relevant for coding in cortex.

**Figure 3:**
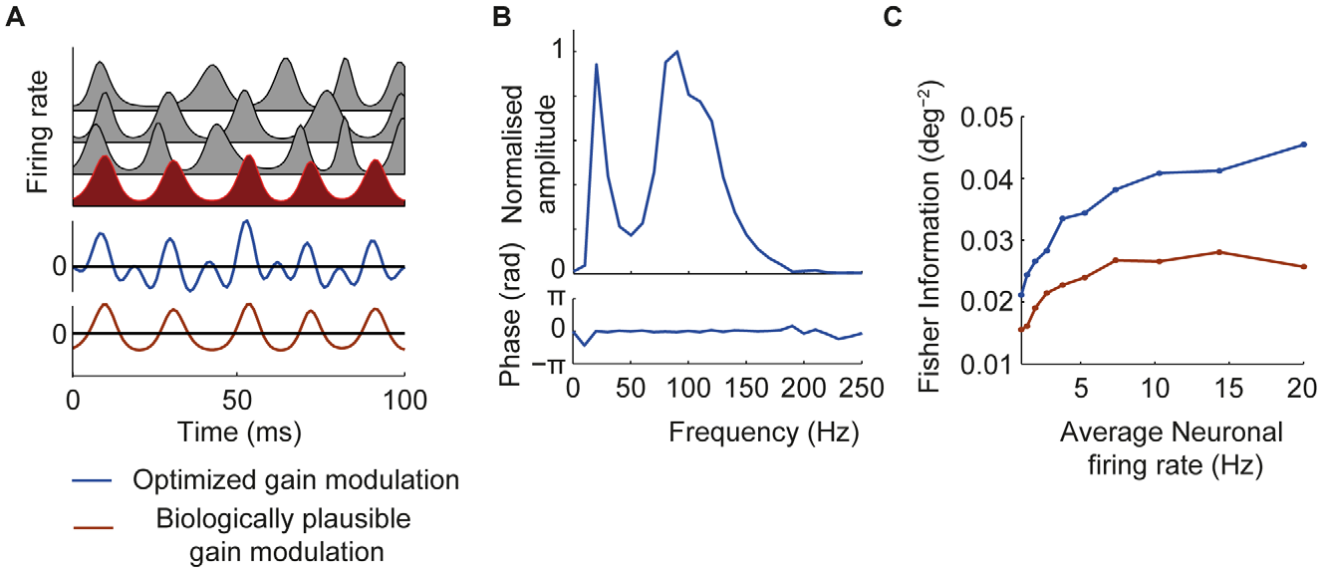
Comparison of optimized and biologically plausible gain modulations. (A) Blue trace is the gain modulation generated by the optimized receiving network; brown trace is a gain modulation that oscillates around zero with the same waveform as the firing rate modulation of the target. (B) Frequency response of the filter that transforms the firing rate modulation of the target input into the optimized gain modulation. (C) Comparison of Fisher information for receiving networks using optimized and alternative gain modulations.

Gain modulations generated by the receiving network in the incoherent distractors condition were often very different in shape from the firing rate modulation of the target (Figure 3A, 6B). This is because the optimized frequency response of the filter that transformed the firing rate modulation of the target input into the gain modulation strongly emphasized the high and low frequency components of the target modulation (Figure 3B). Because such gain modulation may be biologically implausible, we also evaluated the performance of a receiving network that applied a gain modulation that oscillated around 0 with the same waveform as the firing rate modulation of the target input (Figure 3A). This considerably reduced decoding accuracy, resulting in ∼40% lower Fisher information than the optimized receiving network (Figure 3C).

We next tested whether separating distracting inputs from the target in frequency improved performance (Figure 2C,F–H). Because the Von Mises modulations used in the rest of this study contain harmonics which broaden the frequency band occupied by the oscillation, we additionally considered input networks where the firing rate modulation was a sinusoidal function of oscillation phase (Figure 2H). As in other simulations the frequencies of these sinusoidal modulations were allowed to fluctuate from cycle to cycle around their means.

Decoding accuracy increased steeply as the average modulation frequency of the distracting inputs was moved to either higher or lower frequencies than that of the target input (Figure 2H). When distracting inputs were well separated in frequency from the target, decoding accuracy was comparable to that for asynchronous distracting inputs, and Fisher information increased linearly with input network firing rate (Figure 2G). For Von Mises modulations accuracy was reduced when the distractors' modulation frequency was half that of the target (Figure 2F,H), as a result of interference between the first harmonic of the distractors' modulation and the fundamental frequency of the target modulation (see spectra, Figure 2I). We compared Fisher information as a function of distractor frequency for narrowband oscillations, in which cycle-to-cycle frequency fluctuations were small, and for broadband oscillations, in which such fluctuations were large. With broadband oscillations the target and distractor inputs had to be more widely separated in frequency to avoid interference (Figure 2H) as the modulations occupied a broader frequency band (Figure 2I).

Separation in frequency works because sinusoids of different frequency are orthogonal under the overlap integral operation that separates target from distracting inputs. Distracting inputs that are well separated from the target in frequency therefore only contribute noise due to stochastic spiking and not due to fluctuations in the overlap of their firing rate modulation with the gain modulation.

We next explored whether separating inputs in phase allowed the target signal to be accurately separated from distractors. Phase coding, in which assemblies of neurons fire at different phases relative to a global oscillation, has been reported in several neural systems [26]–[30], most notably in the hippocampus where place cells representing past, present and future locations fire at progressively later phases with respect to the theta oscillation.

We evaluated how accurately gain modulation could separate a target input from distractors oscillating coherently with it, evenly separated in phase (Figure 2D). For strongly synchronized activity, decoding was highly accurate, with better performance than for asynchronous distractors (Figure 2F). This was because the absolute value of the gain modulation was small except at the phase where the target but not distracting inputs were firing strongly, reducing noise due to stochastic spiking of distracting inputs.

Performance dropped rapidly as modulation strength was decreased, such that for weakly and moderately synchronized inputs decoding accuracy was worse than for asynchronous distractors. Unsurprisingly, increasing the separation in phase between the peak firing of target and distractor inputs relaxed the degree of synchronization required to reach a given decoding accuracy (data not shown).

### Relationship between target modulation frequency, integration time and accuracy

**Figure 4:**
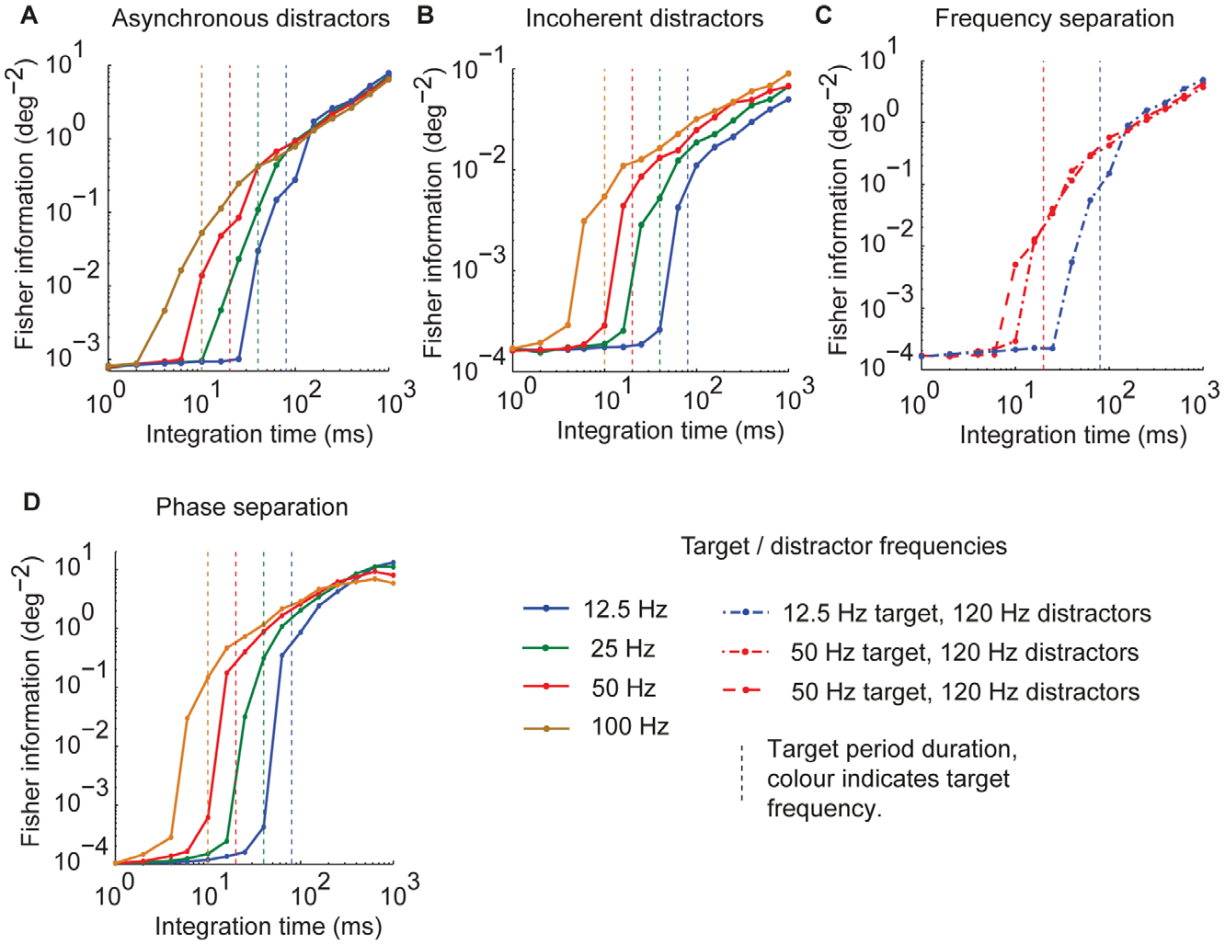
Integration time, modulation frequency and communication accuracy. (A–D) Fisher information as a function of integration time. Target modulation frequency is indicated by line color (see key). For incoherent distractors (A) and phase separation (D) conditions, distractor frequency was the same as target frequency. For frequency separation condition (C), distractor frequency is indicated by line style (see key). The duration of one period of the target input modulation is indicated by the vertical dashed lines, color coded by target modulation frequency.

We examined how changing the duration over which the output of the receiving network was integrated affected the accuracy of selective communication. In the asynchronous distractors condition, Fisher information increased linearly with integration time as long as the integration time was greater than approximately twice the period of the target input modulation (Figure 4A). Below this threshold integration time decoding accuracy dropped precipitously. For efficient selective communication, the integration time must be sufficiently long relative to the target input oscillation period to ensure that the contribution of the target input to the integrated output is minimally affected by the phase of the target modulation, and to ensure that the contribution of the distracting inputs consistently averages to zero over the integration window.

In the incoherent distractors, phase separation, and frequency separation conditions (Figure 4B–D), decoding accuracy also dropped dramatically when the integration time was reduced below approximately two cycles of the target input modulation. In the frequency separation case with target and distractors well separated in frequency, the target but not distractor modulation frequency determined the minimum integration time required.

This frequency dependent minimum integration time required to achieve efficient communication has implications for how different signals may be distributed across different frequency bands. Low modulation frequencies require long integration times, and hence are not suitable for encoding signals that vary on a rapid timescale, whereas higher frequency modulations permit shorter integration times and hence can encode signals that vary on a shorter timescale. This suggests a possible principle contributing to the division of labor between different frequency bands of neural oscillations.

### Bottom-up coherence

In the model considered hitherto, an external control input imposed coherence between the firing rate modulation of the target input network and the gain modulation in the receiving network. An alternative approach to generating coherence would be for the interneuronal circuitry generating the gain modulation to entrain directly to the combined input in a ‘bottom-up’ fashion. Specifically, if no distracting inputs oscillate in the same frequency band as the target, a resonant interneuronal circuit with band-pass characteristics may be able to filter the modulation of the target signal from the combined input activity and use this to generate the appropriate gain modulation.

**Figure 5:**
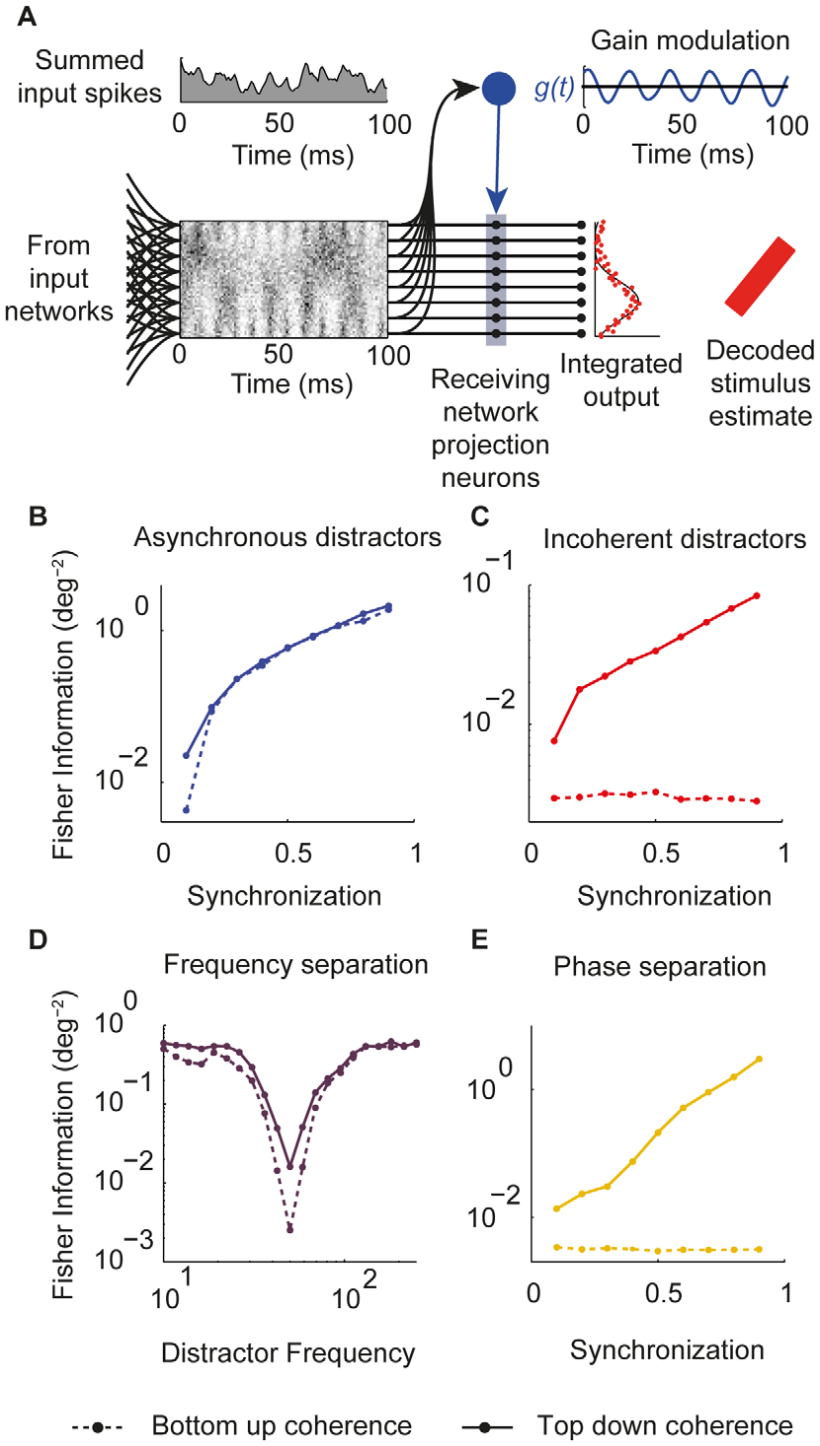
Bottom-up coherence. (*A*) Diagram illustrating receiving network in which gain modulation is a filtered version of the summed combined spike input. (*B*–*E*) Comparison of Fisher information of decoded stimulus estimates for original ‘top-down’ model (solid lines) and ‘bottom-up’ model (dashed lines).

To explore this possibility we considered an alternative version of the model (Figure 5A). Rather than receiving an external control input, the circuitry generating the gain modulation received an input that was simply the summed spiking activity of all input networks. As in the original model, this was linearly filtered to produce a temporal pattern of gain modulation that was applied to the receiving network projection neurons. As before, we optimized the filtering such that the resulting gain modulation best separated target from distracting inputs, and evaluated the performance of the network by integrating the output for 100 ms and decoding the resulting spatial pattern to estimate the target stimulus.

When distracting inputs were asynchronous (Figure 5B), or well separated from the target in frequency (Figure 5D), the performance of the bottom-up model was comparable with that of the top-down model. However, when distracting inputs oscillated in the same frequency band as the target, either incoherently with it (Figure 5C) or at different phases (Figure 5E), the bottom-up model was unable to selectively propagate information about the target input. This was because, with no reference signal to provide information about the phase of target input modulation, there was no way for the receiving network to differentiate between target and distracting inputs.

This bottom-up configuration removes the need for a synchronizing input to the receiving network by effectively hard-wiring in a frequency preference, such that it will respond only to inputs modulated at the correct frequency. Implementing flexible communication would however still require some control circuitry, either to manipulate the dynamical state of the input networks such that only the target oscillates in the pass band, or to shift the resonance of the receiving network to read out signals encoded at different frequencies.

### Arbitrary gain modulations

So far we have presented results for receiving networks which applied a gain modulation that was a linearly filtered version of the firing rate modulation of the target input. We asked if the performance could be improved by allowing the gain modulation to be an arbitrary function of the modulation of the target input. To do this, we took a single instance of the target firing rate modulation over the integration window and used gradient descent to find the pattern of gain modulation that maximized decoding accuracy for this particular target input waveform (see Materials and Methods). Only the modulation of the target input was frozen; distracting input modulations varied as before for each sample in the training and test set. We repeated this for 100 individual instances of the target firing rate modulation, each of which was different because of random variation in its frequency and amplitude and phase.

**Figure 6:**
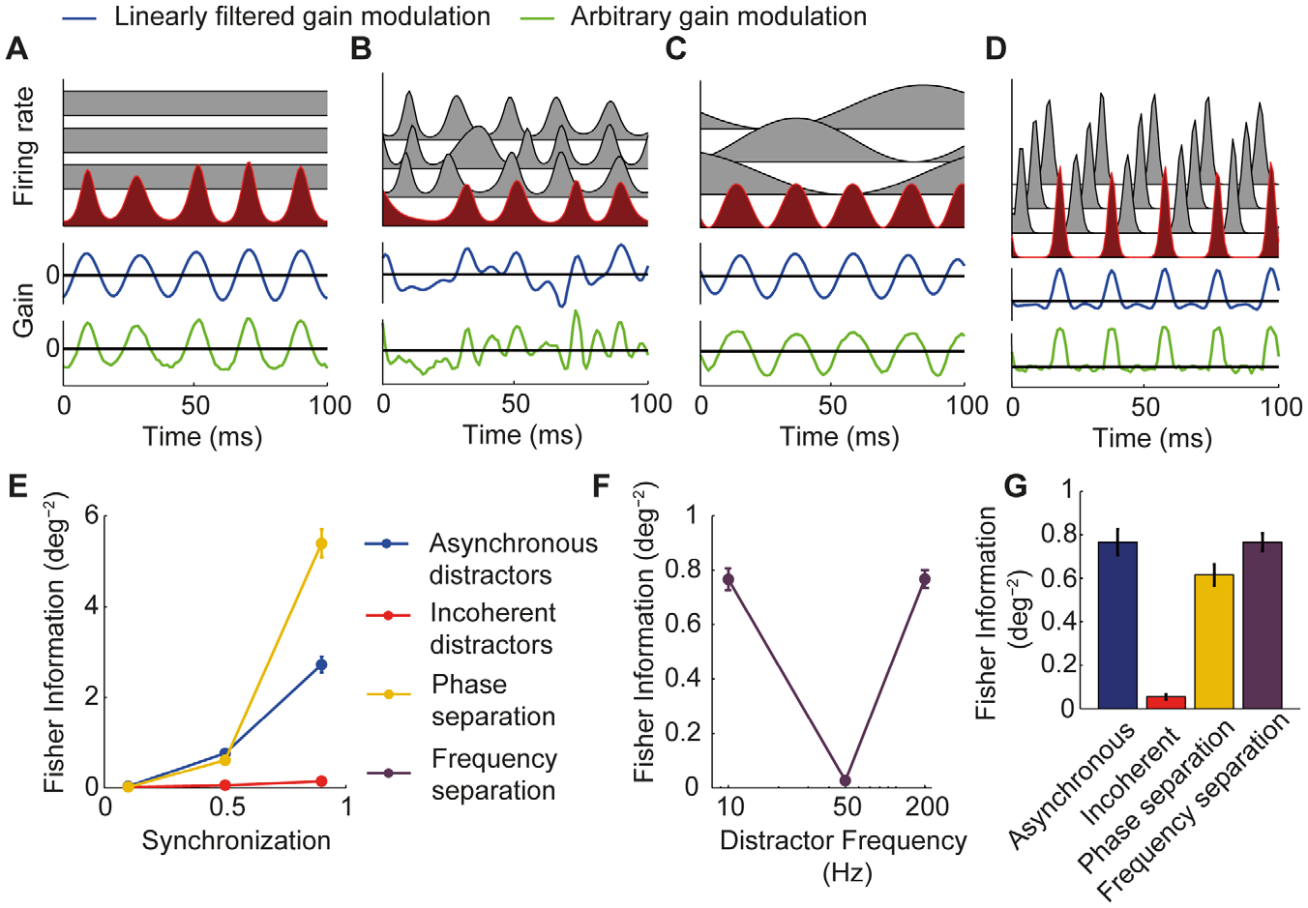
Filtering with arbitrary gain modulations. (A–D) Example input firing rate modulations, gain modulations generated by optimized linear filter (blue trace), and gain modulations found to optimize decoding accuracy for specific examples of target firing rate modulation (green traces). (E) Effect of synchronization strength on decoding accuracy for asynchronous distractors (blue), distractors oscillating incoherently in the same frequency band as the target (red) and distractors oscillating coherently with the target but equally space in phase (yellow). (F) Effect of distractor frequency on decoding accuracy. (G) Comparison of decoding accuracy for different distractor conditions indicated by color as above for synchronization strength of 0.5 and average neuronal firing rate of 5 Hz.

The shape of the gain modulations found by this approach was similar to that found by optimized linear filtering of the target firing rate modulation (Figure 6A–D). For asynchronous distractors (Figure 6A) and those well separated from the target signal in frequency (Figure 6C), gain modulations were close to sinusoidal with a mean value of zero. For distracting inputs oscillating incoherently in the same frequency band as the target, the optimized gain modulation again strongly emphasized frequency components above and below the central frequency of the target firing rate modulation (Figure 6B).

Allowing the gain modulation to be an arbitrary waveform did not qualitatively change the results. As before, the degree of synchronization strongly affected decoding accuracy, with weak modulation resulting in low Fisher information (Figure 6E). Distracting inputs oscillating incoherently in the same frequency band as the target severely compromised accuracy when compared with asynchronous distractors (Figure 6E,G). When distractor and target modulations were well separated in frequency, decoding accuracy was comparable to when distractors were asynchronous (Figure 6E,F).

## Discussion

This study provides a quantitative assessment of the proposal that selective communication can be achieved by coherence between firing rate modulation in a sending region and gain modulation in a receiving region [5]. Our results demonstrate that this is a viable mechanism for gating functional connectivity, potentially allowing robust routing of population-coded information in convergent pathways. However, they show a strong and previously unrecognized dependence of the accuracy of information transmission on the structure and strength of oscillatory activity across a set of inputs.

Our findings question the proposal that incoherent oscillations functionally decouple anatomically connected regions. While random variation in the phase between a firing rate modulation and a gain modulation can reduce the average gain for an input to arbitrarily low levels, this is achieved at the cost of large fluctuations in gain from cycle to cycle. Unless firing rates are very low, these fluctuations are the dominant source of noise in the recovered signal, and severely limit the fidelity with which information encoded by the target input can be recovered. These random fluctuations can be greatly reduced if distracting inputs are asynchronous, or separated from the target input in frequency or phase; these more structured arrangements for multiplexing population codes permit selective communication with much lower noise and higher accuracy. The fundamental reason for this is that where inputs are distinguished by the frequency, phase or amplitude of their oscillations, patterns of gain modulation exist which are strongly driven by the target input but consistently orthogonal to distracting inputs. This is not the case for distracting inputs oscillating incoherently in the same frequency band, in which case much greater interference occurs between the signals.

As we have only considered receiving networks that use multiplicative linear gain modulation, we cannot completely rule out the possibility that a network implementing a more complex operation could more accurately separate signals oscillating incoherently in the same frequency band. If we wish to retain the basic mechanism of coherence between firing rate and gain modulation, an obvious extension is to consider a class of models in which the instantaneous input-output relationship of the receiving network is a non-linear function of the input, and the gain modulation acts by changing the shape of this nonlinearity. We have explored the performance of several models in this class, including those with threshold-linear, power law and threshold-power law nonlinearities (data not shown). In these experiments the gain modulation could vary both a linear input gain and the threshold and/or exponent of the non-linearity. These extensions to the model did not, however, result in any improvement in performance over the linear gain modulation outlined above. Although we cannot claim to have exhaustively explored all possible models in this class, we think it is unlikely that any approach based on coherence between firing rate and gain modulations can efficiently separate signals oscillating incoherently in the same frequency band.

Though it has not been conclusively demonstrated that oscillations play a causal role in controlling functional connectivity, if this hypothesis is correct, the requirement to avoid interference between signals oscillating with different modulations is a probable organizing principle for the richly structured oscillatory activity observed in the mammalian brain. Such activity spans several orders of magnitude in frequency [31], and in several brain regions the phase of firing is actively modulated relative to a single coherent oscillation [26], [28], [29]. These data suggest that the brain can indeed exploit phase and frequency separation to minimize interference between oscillatory signals.

Our findings can help identify whether observed task-dependent changes in oscillatory activity *in vivo* are consistent with a causal role in controlling effective connectivity. Signals must be differentiated by the frequency, phase or amplitude of their modulation to be efficiently separated by coherent gain modulation, so task-dependent changes in these aspects of oscillation structure are plausible signatures of oscillatory control of effective connectivity. Conversely, changes in coherence, i.e. the consistency of a phase relationship, alone do not efficiently support changes in effective connectivity by this mechanism, and hence in the absence of other changes in the structure of oscillatory activity are more likely to be a consequence rather than a cause of changes in signal flow. Striking bursts of transient task dependent oscillatory activity are well documented in many brain regions including in motor cortex during movement preparation [32], [33], the basal ganglia during cue utilization [34], and in visual cortex during working memory [35]. These transient increases in oscillation amplitude may reflect mechanisms for transiently and selectively enhancing effective connectivity between those networks participating in the oscillation event. Various studies have reported switching between distinct oscillation frequencies in a local network [36]–[38], potentially reflecting participation in distinct large scale networks utilizing different frequencies for communication. Systematic changes in the phase of neurons relative to the hippocampal theta rhythm have been observed both within the hippocampus [39] and in extra-hippocampal regions [28], [29]. It is unclear whether these phase shifts should be thought of as a phase code operating in parallel to and separate from rate coding, or whether they are a mechanism for multiplexing multiple firing rate population codes into distinct phases as considered here, allowing functional interactions (or plasticity, see below) between assemblies to be controlled through changes in relative phase. A further interesting example of phase separation was recently identified in the projection from olfactory bulb, where activity in the spatially overlapping projections made by mitral and tufted cells is segregated into opposite phases of the sniff cycle, creating putative independently accessible information channels to cortex [30].

Changes in oscillatory activity dependent on visual spatial attention [3], [16], [40], [41] have been proposed to underlie the selective processing of behaviorally relevant stimuli [5], [7]. Multisite electrocorticographic (ECoG) recording in primates was recently used to evaluate oscillatory synchronization simultaneously in two V1 regions representing separate visual stimuli and a V4 region receiving converging input from these areas [41]. These data provide a detailed description of attention-related changes in gamma oscillations in a convergent pathway during stimulus selection. They show a small (∼4%) increase in oscillation frequency of the V1 network representing the attended stimulus over the V1 network representing the unattended stimulus, comparable gamma amplitude in both V1 regions, and a striking increase in gamma coherence between the attended V1 network and V4. Whether this activity is compatible with the constraints we have identified depends on whether a consistent phase relationship occurs between the two V1 sites, an aspect of the activity not directly explored in the paper. If the phase relationship between the V1 sites is random, variability in the phase between the unattended V1 site and V4 would act as a substantial source of ‘overlap’ noise, limiting the accuracy of selective communication. One possibility discussed by the authors is that theta frequency resetting of gamma oscillation phase [42] across V1 and V4, combined with the frequency offset between the V1 networks, creates periods in which the attended V1 site consistently leads the unattended site. The consistent phase offset produced in such an arrangement could be efficiently exploited for selective communication. Further analysis will be needed to establish whether such structured activity is in fact generated across the V1 networks during attention.

We note that gamma oscillations in V1 are particularly amenable to experimental phase manipulation as they are readily entrained by flickering visual stimuli [43], as expected given the response dynamics of gamma oscillating networks in vitro [44]. A recent study found no effect of manipulating the relative phase of gamma frequency flicker between target and distracting stimuli on selective attentional processing [45], although without concurrent electrophysiological data it is unclear how effectively cortical activity was manipulated. The combination of flicker manipulations with ECoG recordings is a potentially powerful way of testing the functional importance of attention dependent changes observed in V1–V4 gamma coherence.

Our data indicate that the strength of oscillatory modulation of the target signal critically determines accuracy of selective communication and hence can serve as another important clue in evaluating whether *in vivo* oscillatory phenomena play a causal role in controlling effective connectivity. Weak oscillations result in poor signal-to-noise ratios because the firing rate modulation read out by the receiving network is small relative to noise from stochastic spiking of individual neurons. This conclusion is likely to generalize beyond CTC to other mechanisms in which the principal carrier of information is non-zero frequency components of the firing rate generated by oscillatory network activity [15].

Estimating the modulation depth of sparsely synchronized oscillatory activity is technically challenging. Individual neurons fire irregularly at rates potentially well below the oscillation frequency, such that the spike pattern of a single neuron provides little information about the population firing rate modulation. The widely used measures of spike-field and spike-spike coherence do not map directly onto modulation strength as they are confounded by firing rate [46], which substantially impedes attempts to evaluate modulation strength from much of the published literature. A common approach to estimating modulation strength is to look at the distribution of spikes relative to the phase of a band-pass filtered local field potential (LFP) oscillation. This method can underestimate modulation strength if the LFP signal is corrupted by noise, for example from neurons not participating in the oscillation, or if the analysis combines activity from periods with and without strong oscillation. Despite these technical difficulties reported spike phase histograms show a wide range of modulation strengths across different oscillations, from very strong modulations during hippocampal theta oscillations [47], and oscillations in the olfactory system of zebrafish [27] and locusts [48], to apparently weaker modulation in some studies of gamma oscillations in the hippocampus [49], [50] and entorhinal cortex [49]. Our results suggest that, where oscillations are genuinely weak, mechanisms exploiting them for selective routing of signals would recover only a tiny fraction of the information represented in the sending population.

Our use of a highly simplified non-biophysical model in this work was necessary to permit model optimization and hence to find an upper bound on how accurately coherent gain modulation could separate target from distracting inputs. However, it raises the question of whether a biological network or biophysical model could achieve this performance. A biophysical implementation of the receiving network must generate approximately multiplicative gain modulation coherent with either a top-down control input, or with a particular frequency component of the combined input in a bottom-up fashion. Several biophysical mechanisms including shunting inhibition and synaptic noise are known to produce approximately multiplicative gain modulation in individual neurons [20], [21]. Entrainment of oscillatory or resonant local circuitry in the receiving network is a plausible mechanism for generating the required temporal patterning of gain modulation. We recently demonstrated that the dynamical properties of gamma oscillations in the CA3 region support entrainment to periodic inputs [44], though the consequences of such entrainment for the gain of signal transmission remain to be established. These data suggest that entrainment phase may be controlled by varying the natural frequency of the network relative to the input, or by varying the relative coupling of the input to excitatory and inhibitory populations.

We previously developed a biophysical model [15], which exploits network resonance effects at the boundary between asynchronous and oscillating states [51] to selectively respond to inputs oscillating at a specific frequency. While the biophysical model implemented a similar functionality to the ‘bottom-up’ coherence configuration considered here, there are some key differences in the mechanism of operation that bear outlining explicitly. In both models, information was represented in the input networks as spatial patterns of firing rate, while the target input was differentiated from distractors by multiplicative modulation (here represented explicitly, in the biophysical model generated by sparsely synchronized network dynamics). Both models exploit the fact that multiplicative modulation selectively reproduces the spatial pattern of firing rate into those higher frequency components of the firing rate present in the modulation [15]. However, the models differ in the way the receiving network reads out the resulting spatial patterns of firing rate oscillation. The biophysical model essentially converts the amplitude of input firing rate oscillation at a given frequency into the average firing rate of the output neurons through a process of bandpass filtering followed by half-wave rectification. The bandpass filtering is implemented by a combination of resonant feed forward inhibition and synaptic filtering which ensures that the net input current received by the output neurons is a bandpass-filtered version of the input activity. The spike threshold then rectifies this input current to produce an output firing rate. In the current model, readout is by multiplicative gain modulation followed by integration over time, exploiting the orthogonality of different frequency components under overlap integration to separate target from distracting signals. Thus although the previous biophysical model utilizes a similar coding strategy to the current model and achieves similar functionality to the ‘bottom up’ configuration, the mechanisms underlying the filtering in the two models differ significantly.

Implementation of biophysical models that operate on the same principle as the current model is a clear direction for future work. Several studies working in this direction [12], [14], [52], [53] have demonstrated some degree of input selectivity on the basis of modulation, particularly for phase separated inputs [52], [53]. Our understanding is that the selective communication performance achieved by these biophysical models is substantially lower than the current optimized model. Further work is required to establish how efficiently and robustly biophysical networks can utilize coherent gain modulation to extract information multiplexed into patterns of firing rate modulation, and what network architectures are effective in this task.

Throughout this work we have discussed gain modulation that acts on the input-output relationship for the activity of a population of neurons. Oscillatory activity can also modulate the gain of synaptic plasticity [54], [55], and spike timing dependent plasticity with an oscillating post synaptic population will also produce periodic modulation of the gain for plasticity. Periodic modulation of the gain for plasticity coherent with the firing rate modulation of a target input could selectively enhance plasticity for that input just as coherent modulation of neuronal input-output gain can permit selective response to a target input. As our results are due to signal to noise considerations they are equally applicable to identifying which structures of activity permit accurate selective plasticity of a subset of inputs by oscillatory modulation of the gain for plasticity.

In conclusion, accurate and selective communication can be achieved by coherence between gain and firing rate modulations. However, to achieve a high signal to noise ratio the oscillatory modulation of the target signal must be strong, and distracting inputs must be distinguished from the target by frequency, phase or amplitude of oscillation. Failure to satisfy these constraints greatly reduces the accuracy of information transmission. Where oscillatory activity plays a causal role in modulating functional connectivity we expect it to be organized to maximize the accuracy of signal propagation.

## Supporting Information

Figure S1 **Incoherent distractors not broadband target degrade communication accuracy.** (*A*–*B*) Example firing rate modulation of the target (red) and distracting inputs (gray) over the 100 ms integration time. (C) Fisher Information as a function of input network firing rates. Condition indicated by color of trace as shown in key.
(TIF)

Figure S2 **Low firing rate threshold.** (*A*) Variance of noise in integrated output of receiving network due to an asynchronous (solid line) or an oscillating (dotted line) distracting input as function of the mean firing rate in the distracting inputs. (*B*) Ratio of the noise variances plotted in (*A*); vertical line indicates firing rate threshold at which ratio is two. (*C*) As for (*A*) but for different integration times and modulation frequencies indicated by line color (see key). (*D*) as for (*B*) but with population firing rate expressed in spikes per cycle of oscillation. (*E*) Firing rate threshold plotted as a function of the synchronization strength of the oscillating distracting input for optimized and alternative ‘biologically plausible’ gain modulation (See Fig. S2).
(TIF)

Text S1 **Low firing rate threshold.**
(DOCX)
