# Supplementary figures and images for: Efficient “Communication through Coherence” Requires Oscillations Structured to Minimize Interference between Signals
Source: PLoS Comput Biol. 2012 Nov 8;8(11):e1002760. doi: 10.1371/journal.pcbi.1002760 (PMC3493486; doi:10.1371/journal.pcbi.1002760)

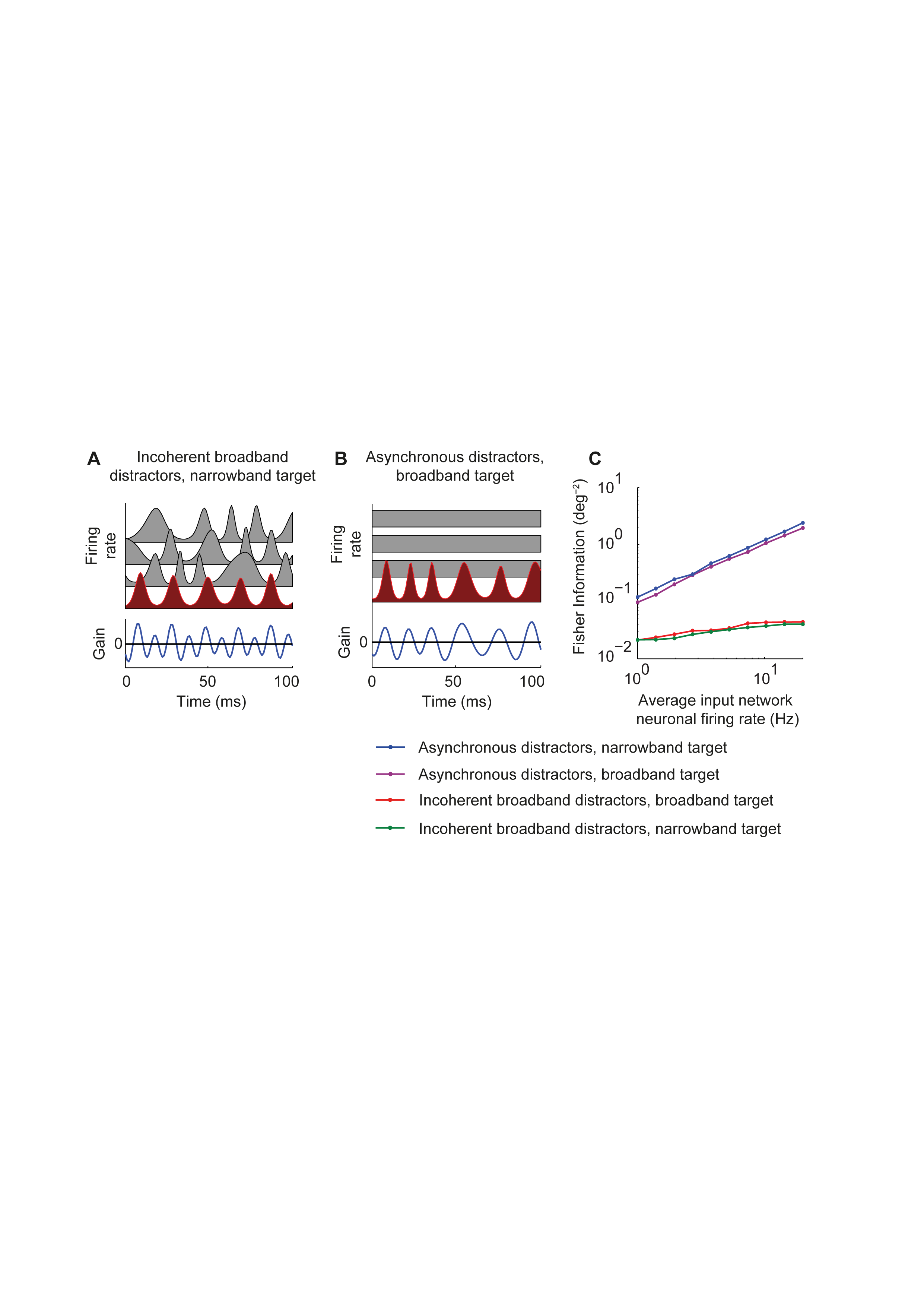

Supplement: Figure S1 — Incoherent distractors not broadband target degrade communication accuracy. (A–B) Example firing rate modulation of the target (red) and distracting inputs (gray) over the 100 ms integration time. (C) Fisher Information as a function of input network firing rates. Condition indicated by color of trace as shown in key. (TIF) [file pcbi.1002760.s001.tif]

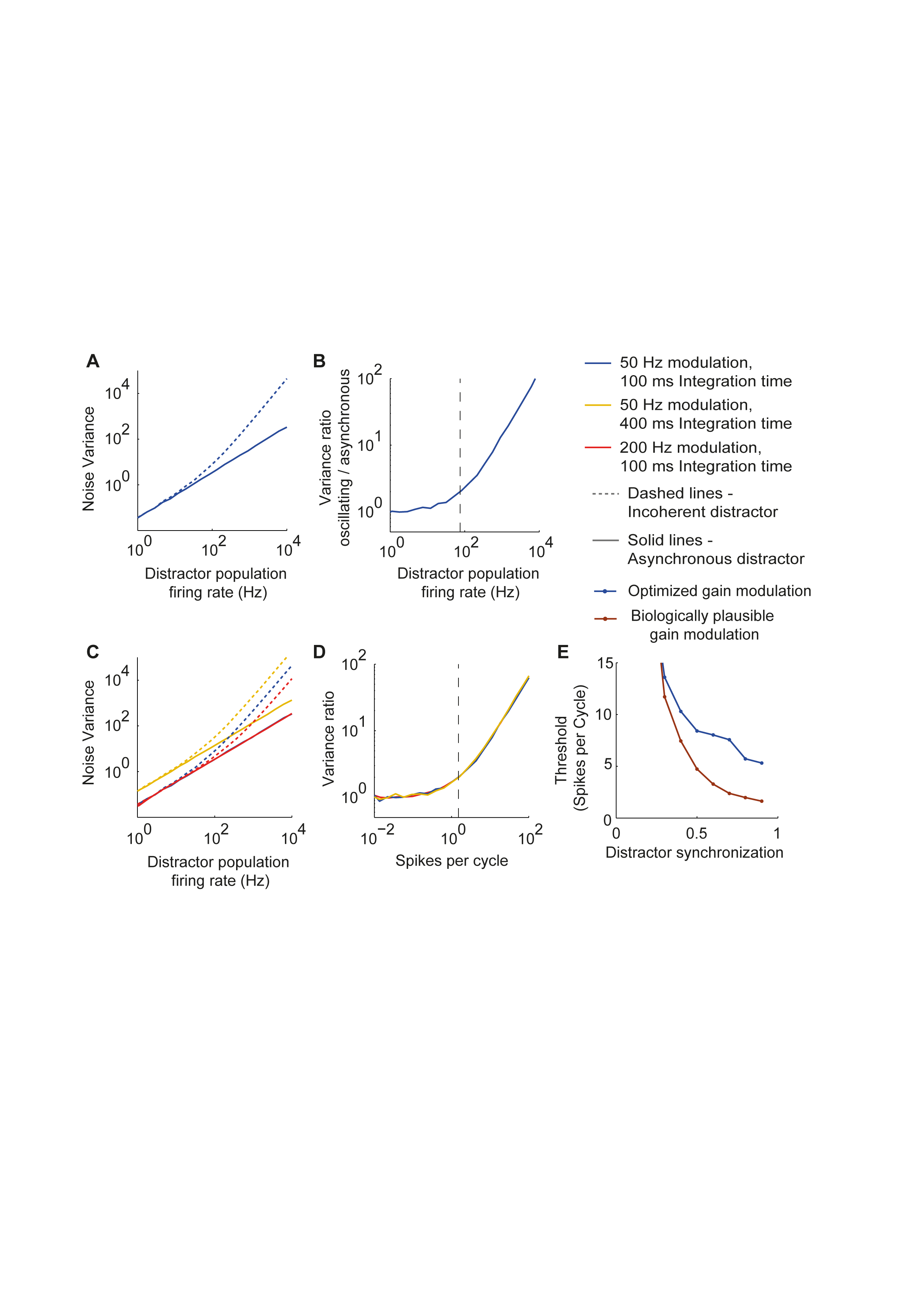

Supplement: Figure S2 — Low firing rate threshold. (A) Variance of noise in integrated output of receiving network due to an asynchronous (solid line) or an oscillating (dotted line) distracting input as function of the mean firing rate in the distracting inputs. (B) Ratio of the noise variances plotted in (A); vertical line indicates firing rate threshold at which ratio is two. (C) As for (A) but for different integration times and modulation frequencies indicated by line color (see key). (D) as for (B) but with population firing rate expressed in spikes per cycle of oscillation. (E) Firing rate threshold plotted as a function of the synchronization strength of the oscillating distracting input for optimized and alternative ‘biologically plausible’ gain modulation (See Fig. S2). (TIF) [file pcbi.1002760.s002.tif]
