# Supplementary material for: Efficient “Communication through Coherence” Requires Oscillations Structured to Minimize Interference between Signals
Source: PLoS Comput Biol. 2012 Nov 8;8(11):e1002760. doi: 10.1371/journal.pcbi.1002760 (PMC3493486; doi:10.1371/journal.pcbi.1002760)
Supplement: Text S1 — Low firing rate threshold. (DOCX) [file pcbi.1002760.s003.docx]

**Supporting Information:**

**Text S1: Low firing rate threshold**

When distracting inputs oscillate incoherently in the same frequency band as the target signal, they generate noise in the output of the receiving network because of random variation in the overlap between their firing rate modulation and gain modulation in the receiving region. This overlap noise can be minimized by structured oscillatory activity in which distracting inputs are asynchronous or separated from the target in phase or frequency. The relative advantage of such structured activity increases with the firing rate of the input networks because, as the firing rate increases, noise due to stochastic spiking of individual neurons becomes smaller relative to the signal size while the relative size of overlap noise is unchanged. This suggests that there is a firing rate threshold below which structured oscillatory activity offers little advantage. We show here that such a firing rate threshold exists but argue that it is low relative to firing rates relevant to coding in cortex.

Defining a firing rate threshold for a population code is complicated as the code is itself a distribution of firing rates over neurons. To provide a number that is straightforward to interpret we considered a local region of receiving network for which all afferents have the same stimulus tuning, such that each input is characterized by an average population firing rate $R^{n}$ and modulation $m^{n}(t)$. The spiking activity due to input network N is:

$$s^{n}\left(t\right)\sim Poisson(R^{n}m^{n}\left(t\right))$$

The total spike input is:

$$s(t)=\sum_{n} s^{n}(t)$$

The output of the receiving network is:

$$O(\text{t})=g\left(t\right)s\left(t\right)$$

Where $g\left(t\right)$ is the gain modulation of the receiving network. The integrated output of the receiving network is:

$$P=\int_{0}^{T} O(\text{t})dt$$

To determine the firing rate threshold at which overlap noise becomes dominant we compared the noise due to an asynchronous distracting input with that due to an incoherently oscillating distracting input (both with the same average population firing rate). This depends on their firing rates, the gain modulation and on the firing rate modulation of the incoherently oscillating input. As in Figure 2B, we assume that the target input and the oscillating distracting input have broadband oscillations with the same central frequency and same synchronization strength. We considered two different gain modulations; that found in Figure 2 to maximize decoding accuracy for incoherently oscillating distractors (but which is physiologically implausible as it is often a multimodal function of oscillation phase), and the biologically plausible gain modulation used in Figure 3, which oscillated about zero with the same waveform as the firing rate modulation of the target input.

For networks in which the average value of the gain modulation over time is zero, either an asynchronous or an incoherently oscillating distracting input contributes on average zero to the integrated output, irrespective of its average firing rate. However, for any given time window the contribution will not be exactly zero because of stochastic spiking of individual neurons and variability in the overlap between the firing rate modulation of the distracting input and gain modulation in the receiving network. Across an ensemble of time windows there will be a distribution centered on a mean of zero. The variance of this distribution is a measure of the noise contributed by the distracting input:

$$Distractor noise variance= var ( \int_{0}^{T} g\left(t\right)s^{n}(t)dt )$$

We evaluated the noise variance as a function of average population firing rate for the asynchronous and incoherently oscillating distracting inputs (Figure S2A). For the asynchronous distracting input noise variance increased linearly with average firing rate as expected for Poisson noise. However, for the distracting input oscillating incoherently in the same frequency band as the target signal the relationship between firing rate and variance was different at low and high firing rates. At low firing rates the noise variance was identical to that due to an asynchronous distractor and increased approximately linearly with firing rate (Figure S2A). At higher firing rates the noise variance for the oscillating distractor increased with the square of the firing rate, rapidly becoming much larger than that due to the asynchronous distractor. In the high firing rate regime the dominant cause of noise is random variability in the overlap between firing rate modulation of the input and gain modulation in the receiving region.

We defined a threshold that distinguished these regimes as the population firing rate at which the noise due to the oscillating distractor was two-fold larger than that due to the asynchronous distractor (Figure S2B). This threshold was invariant to changes in the integration time, as noise variance for both asynchronous and oscillating distractors scaled proportionally (Figure S2C). The firing rate threshold increased linearly with the oscillation frequency because at higher frequencies the overlap between gain modulation and firing rate modulation was averaged over more oscillation cycles (Figure S2C). By expressing the firing rate threshold in units of spikes per cycle rather than Hz, the threshold remained invariant to changes in the oscillation frequency (Figure S2D).

The threshold firing rate varied with the synchronization strength of the inputs (Figure S2E). This is because with weak modulations overlap noise is smaller relative to stochastic spiking of individual neurons. However, where target and distracting input have comparable modulation strength, this reflects a worsening of the signal-to-noise ratio due to stochastic spiking rather than an improvement in signal-to-noise ratio due to overlap variability. As shown above, for a given firing rate weak oscillations give much worse decoding accuracy (Figure 2F).

For moderately to strongly oscillating distracting inputs, the threshold population firing rate was in the range 5-10 spikes per cycle for a receiving network using the optimized gain modulation. For the receiving network with a biologically plausible gain modulation the threshold firing rate was in the range 1.5-5 spikes per cycle.

A threshold of 5 spikes per cycle corresponds to a population firing rate of 200-400 Hz for gamma oscillations, and proportionally less for lower oscillation frequencies. Although average firing rates in cortex are thought to be low (< 1 Hz) [1], mean rates in sensory cortex during behavioral tasks are considerable higher (5-10 Hz) [2,3] with cells that are strongly driven by a particular stimulus firing at rates up to 100 Hz. A region of network receiving a distracting input comprised of just a few tens of active neurons would therefore be in the high firing rate regime where ‘non-communication through non-coherence’ performs poorly relative to schemes using more structured oscillations.

**Supplementary References**

1. Lennie P (2003) The Cost of Cortical Computation. Current Biology 13: 493–497. doi:10.1016/S0960-9822(03)00135-0.

2. Vijayan S, Hale GJ, Moore CI, Brown EN, Wilson M (2010) Activity in the barrel cortex during active behavior and sleep. J Neurophysiol 103: 2074–2084. doi:10.1152/jn.00474.2009.

3. O’Connor DH, Peron SP, Huber D, Svoboda K (2010) Neural Activity in Barrel Cortex Underlying Vibrissa-Based Object Localization in Mice. Neuron 67: 1048–1061.
